# Strong Exchange Coupling in a Trimetallic Radical‐Bridged Cobalt(II)‐Hexaazatrinaphthylene Complex

**DOI:** 10.1002/anie.201600694

**Published:** 2016-03-21

**Authors:** Jani O. Moilanen, Nicholas F. Chilton, Benjamin M. Day, Thomas Pugh, Richard A. Layfield

**Affiliations:** ^1^ School of ChemistryThe University of ManchesterOxford RoadManchesterM13 9PLUK; ^2^ Department of ChemistryUniversity of JyvaskalaP.O. Box 3540014JyvaskalaFinland

**Keywords:** cobalt, hexaazatrinaphthylene, magnetism, non-innocent ligands, radicals

## Abstract

Reducing hexaazatrinaphthylene (HAN) with potassium in the presence of 18‐c‐6 produces [{K(18‐c‐6)}HAN], which contains the S=1/2 radical [HAN]^.−^. The [HAN]^.−^ radical can be transferred to the cobalt(II) amide [Co{N(SiMe_3_)_2_}_2_], forming [K(18‐c‐6)][(HAN){Co(N′′)_2_}_3_]; magnetic measurements on this compound reveal an S=4 spin system with strong cobalt–ligand antiferromagnetic exchange and J≈−290 cm^−1^ (−2 J formalism). In contrast, the Co^II^ centres in the unreduced analogue [(HAN){Co(N′′)_2_}_3_] are weakly coupled (J≈−4.4 cm^−1^). The finding that [HAN]^.−^ can be synthesized as a stable salt and transferred to cobalt introduces potential new routes to magnetic materials based on strongly coupled, triangular HAN building blocks.

Redox‐active ligands continue to provide a rich source of fascinating coordination chemistry.^1^ In addition to the fundamental interest in the ability of some ligands to act as electron reservoirs, metal complexes of redox‐active ligands have been developed for a range of applications. Important examples of ligand non‐innocence are found in biological coordination chemistry, with NO, O_2_ and dithiolenes featuring prominently.^2^ The concepts of ligand non‐innocence and metal‐ligand cooperativity have been used to design new catalytic reactions,^3^ and the electron transport properties of complexes with non‐innocent ligands have been harnessed for applications in molecular electronics and redox‐flow batteries.^4^, ^5^ The influence of redox‐active ligands on molecule‐based magnets has also produced striking results, such as room‐temperature magnetic ordering in [V(TCNE)_*x*_⋅*y* CH_2_Cl_2_] (*x*≈2, *y*≈0.5; TCNE=tetracyanoethylene),^6^ and a large coercive field at 11 K in the lanthanide single‐molecule magnet [K(18‐c‐6)][Tb_2_{N(SiMe_3_)_4_(μ‐N_2_)(THF)_2_] (18‐c‐6=18‐crown‐6).^7^

Hexaazatriphenylene (HAT, Figure 1) is the simplest member of a family of electron‐deficient tris(bidentate) ligands with considerable non‐innocent character.^9^, ^10^, ^11^, ^12^, ^13^, ^14^ An area in which the potential non‐innocence of HAT ligands has not yet been exploited is molecular magnetism, principally because stable, synthetically useful radical derivatives of HAT are unknown. HAT‐type ligands are of interest in this context because they can bind metal ions in a triangular arrangement, which can in principle lead to frustrated or non‐collinear spin systems. Studies of HAT‐mediated magnetic exchange are rare and have been limited to the unreduced ligand.^15^, ^16^, ^17^, ^18^, ^19^, ^20^ Although the exchange coupling in M_3_HAT complexes with M=Co^II^, Fe^II^ or Cu^II^ is antiferromagnetic, it is also very weak (|*J*|<2 cm^−1^ for 2 *J* spin Hamiltonians), hence spin‐frustration was not observed.

**Figure 1:**
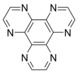
Hexaazatriphenylene (HAT).

We were interested to see if strong exchange could be induced in an M_3_HAT complex by exploiting the non‐innocent character of the ligand. Achieving this aim would introduce possibilities for developing new building blocks for the assembly of molecule‐based magnets. To avoid the use of explosive precursors, we used the derivative hexaazatrinaphthylene (HAN). Our aim was to reduce HAN to its corresponding radical anionic form, and then to transfer [HAN]^.−^ to a transition metal ion. Because HAT‐type compounds suffer from poor solubility, we chose the two‐coordinate cobalt(II) amide [Co(N′′)_2_] (N′′=N(SiMe_3_)_2_) as the transition metal synthon,^21^ which provides anisotropic d^7^ ions and lipophilic trimethylsilyl groups.

Heating potassium, HAN and 18‐c‐6 in toluene produced [{K(18‐c‐6)}HAN], [{K(18‐c‐6)}**1**], as an analytically pure red powder in 90 % yield (Scheme 1). The UV/vis/NIR spectrum of [{K(18‐c‐6)}**1**] in THF consists of a series of broad, overlapping absorptions at *λ*<450 nm, which are probably due to transitions from the π‐HOMOs to the low‐lying π* LUMOs (Figure S3). The spectrum also shows a well‐resolved transition at 590 nm, which is typical for an organic π‐radical and likely corresponds to a SOMO‐LUMO π–π* transition.^22^

**Scheme 1:**
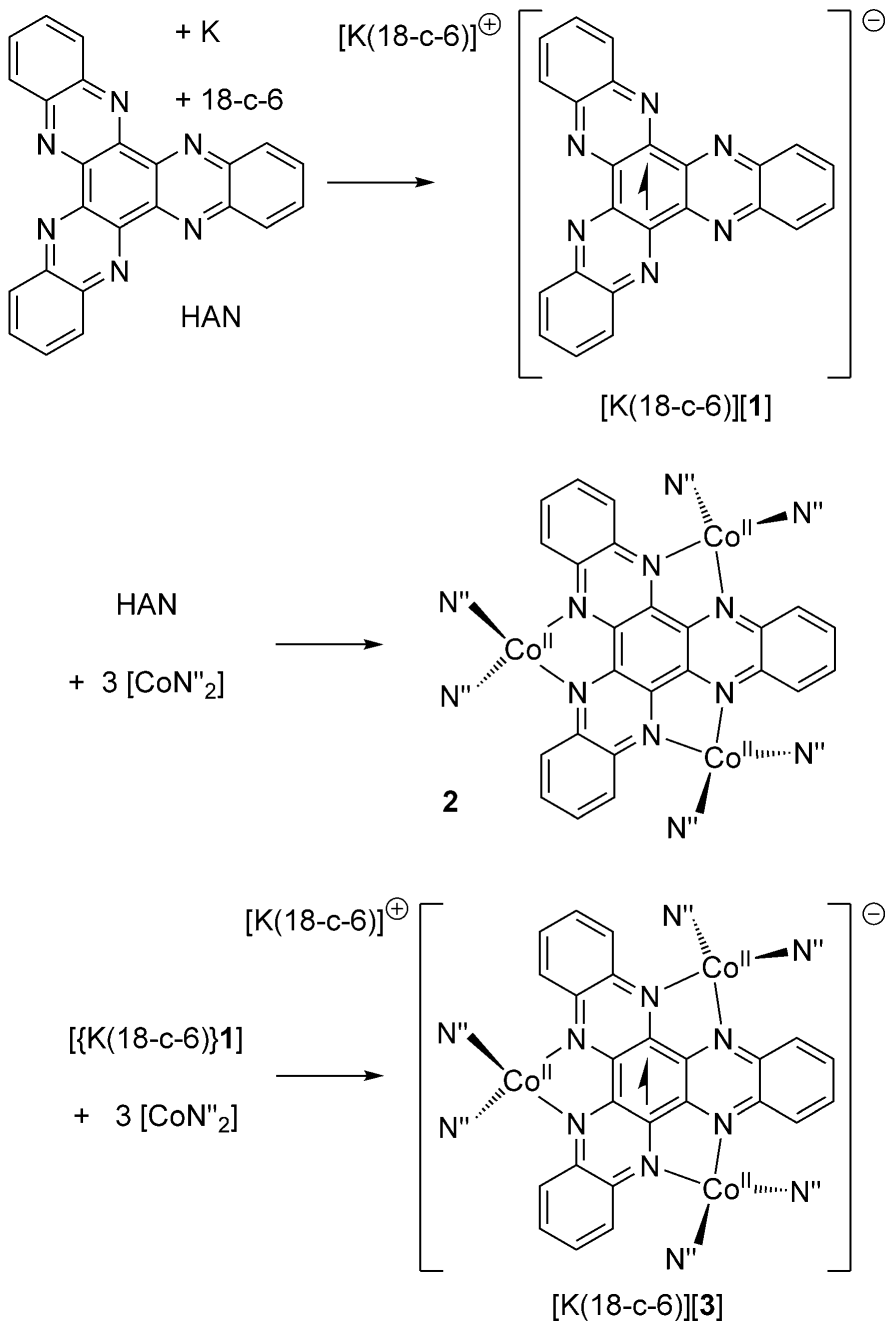
Synthesis of [{K(18‐c‐6)}**1**], **2** and [K(18‐c‐6)][**3**] (N′′= N(SiMe_3_)_2_).

The X‐band EPR spectrum of [{K(18‐c‐6)}**1**] in THF solution at 293 K features a single resonance centred on *g=*2.0033, confirming its radical nature, with extensive hyperfine structure (Figures 2 and S10 in the Supporting Information (SI)). The spectrum was simulated using EasySpin with the hyperfine coupling constants in Table S2. Further insight into the molecular and electronic structure of [{K(18‐c‐6)}**1**] was obtained through DFT calculations owing to the reluctance of the compound to form single crystals. The geometry of [{K(18‐c‐6)}**1**] was fully optimized in the *C*_3_ point group using the B3LYP exchange‐correlation functional in combination either with 6‐311G** or with def2‐TZVP triple‐ζ basis sets (see SI for computational details). For comparative purposes, the same calculations were also carried out on the pristine [HAN]^.−^ radical anion in the *D*_3*h*_ point group. The B3LYP/6‐311G** (B3LYP/def2‐TZVP) structure for [{K(18‐c‐6)}**1**] consists of a near‐planar HAN ring system, with the potassium cation positioned perpendicular to the central C_6_ unit, with K‐C distances to C(1)–C(6) of 3.683–3.684 Å (3.709–3.709 Å) and a distance of 3.385 Å (3.414 Å) from potassium to the centre of the C_6_ ring. The potassium cation is 7‐coordinate by virtue of the π‐bonded HAN ligand and 18‐crown‐6, and is positioned 0.368 Å (0.436 Å) out of the mean plane of the 18‐c‐6 oxygen atoms towards the C_6_ ring. The calculations show that the spin density on the [HAN]^.−^ radical anion **1** (Figure 2) and in [{K(18‐c‐6)}**1**] (Figure S11) are very similar. Significant spin density resides on the nitrogen atoms of [{K(18‐c‐6)}**1**], with the remaining spin density being distributed across the carbon atoms.

**Figure 2:**
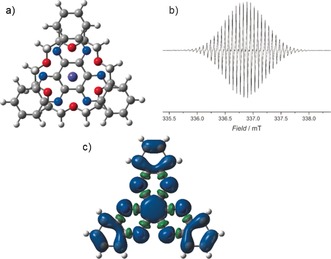
a) Optimized geometry of [{K(18‐c‐6)}**1**] viewed perpendicular to the HAN plane (purple=K; grey=C; blue=N; red=O; white=H). b) Solution‐state X‐band EPR spectrum of [{K(18‐c‐6)}**1**] at 293 K in THF. c) Spin density for **1** (blue=α spin density; green=β spin density, B3LYP/6‐311G**).

The reactions of HAN and of [{K(18‐c‐6)}**1**] with [Co(N′′)_2_] proceeded according to Scheme 1. The addition of [Co(N′′)_2_] to HAN in toluene gave a green solution from which black crystals of [(HAN){Co(N′′)_2_}_3_]⋅toluene (**2**⋅toluene) were grown. The reaction of [Co(N′′)_2_] with [{K(18‐c‐6)}**1**] produced a dark red solution, which allowed black crystals of [K(18‐c‐6)][(HAN){Co(N′′)_2_}_3_]⋅2(toluene), or [K(18‐c‐6)][**3**]⋅2(toluene), to be isolated. The structures of both cobalt‐containing compounds were determined by X‐ray diffraction (Figure 3, Table S1).^23^

**Figure 3:**
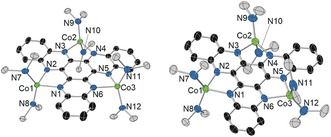
Structures of **2** (left) and **3** (right). Thermal ellipsoids at 50 % probability. For clarity, the methyl groups and the hydrogen atoms are omitted (black=C; grey=Si).

Complexes **2** and **3** have similar molecular structures, with both containing three Co^II^ centres bonded to two nitrogen atoms on the unreduced and reduced HAN ligand, respectively, and to the nitrogen atoms of two amide ligands. In **2**, the cobalt environments are distorted tetrahedral, featuring relatively long Co−N bonds to the HAN ligand in the range 2.063(3)–2.118(3) Å (average 2.094 Å) and relatively short Co−N bonds to the amide ligands of 1.916(4)–1.945(4) Å (average 1.932 Å). The analogous Co−N distances in **3** are 2.081(3)–2.127(3) Å (average 2.099 Å) and 1.960(3)‐1.975(3) Å (average 1.968 Å). The N‐Co‐N angles involving the HAN ligand in **2** are 78.85(13)–79.55(13)°, whereas the other N‐Co‐N angles are much wider at 98.62(14)–136.69(16)°. The N‐Co‐N angles in **3** adopt a similar pattern, that is, 78.94(10)–79.39(10)° and 103.05(10)–128.05(13)°. The C−N bond lengths in **2** and **3** lie in the same range, that is, 1.328(5)–1.386(5) Å (1.355 Å average) and 1.329(4)–1.389(4) Å (1.360 Å average), and the C−C bond lengths are also similar, that is, 1.353(5)–1.437(6) Å (1.401 Å average) and 1.359(5)–1.449(4) Å (1.398 Å average), respectively.

The magnetic susceptibility of both materials was measured in a field of *H*=1 T at *T=*2–300 K (Figure 4). For **2**, the *χ*_M_ *T*(*T*) product declines slowly from 7.19 cm^3^ K mol^−1^ at 300 K to 0.42 cm^3^ K mol^−1^ at 2 K, and shows a slight inflection at about 30 K. The inflection is reproducible across different samples, and is unlikely to be due to torqueing as the samples were restrained in eicosane. The appearance of the *χ*_M_ *T*(*T*) data for **2** suggests weak antiferromagnetic exchange between the cobalt(II) centres. The high‐temperature value of *χ*_M_ *T* is significantly greater than the value of 5.63 cm^3^ K mol^−1^ expected for three non‐interacting *S*=3/2 cobalt(II) ions with *g*‐factors of 2.00, indicating appreciable spin‐orbit mixing with orbitally degenerate excited states in **2**. The *χ*_M_ *T*(*T*) profile for [K(18‐c‐6)][**3**] is markedly different to that of **2**. Cooling the sample from 300 K to 50 K produces a steady increase in *χ*_M_ *T*(*T*) from 9.66 cm^3^ K mol^−1^ to reach a maximum of 12.19 cm^3^ K mol^−1^. This behaviour arises from strong antiferromagnetic interactions between the radical ligand and the three cobalt(II) centres, effectively resulting in a ferromagnetic alignment of the metal‐based spins. At lower temperatures, a rapid decrease in *χ*_M_ *T* is observed, reaching a value of 4.49 cm^3^ K mol^−1^ at 2 K. For [K(18‐c‐6)][**3**], the value of *χ*_M_ *T* at 300 K is very close to the value of 10.0 cm^3^ mol^−1^ K expected for an *S=*4 state with *g=*2.0, arising from strong antiferromagnetic interactions between the *S=*3/2 cobalt(II) centres and the *S=*1/2 [HAN]^.−^ radical.

**Figure 4:**
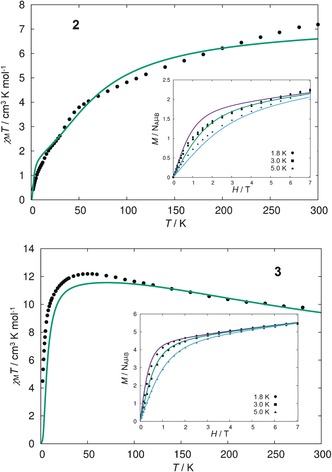
Plots of *χ*_M_ *T*(*T*) in a field of 1 T, and plots of *M*(*H*) (inset) at 1.8 K, 3 K and 5 K, for **2** and [K(18‐c‐6)][**3**]. The dots the experimental data and the solid lines are fits according to the parameters described in the text.

The field dependence of the magnetization for **2** and [K(18‐c‐6)][**3**] was measured at various fields in the range *H*=0–7 T, using temperatures of 1.8 K, 3.0 K and 5.0 K (Figure 4). At 1.8 K, the magnetization for **2** rises gradually to reach a value of *M*=1.45 μ_B_ at 2.0 T and then rises more slowly, without saturating, to reach *M*=2.25 μ_B_ at 7.0 T. In contrast, the magnetization of [K(18‐c‐6)][**3**] increases more rapidly at lower fields, with a much larger magnetization of *M*=4.66 μ_B_ at 1.8 K and 2.0 T, and as the field increases the magnetization approaches saturation with *M*=5.49 μ_B_ at 7.0 T. The reduced magnetisation data for **2** and [K(18‐c‐6)][**3**] show non‐ superimposable isotherms, indicating significant magnetic anisotropy for both complexes (Figure S13).

To quantify the magnetic interactions in **2** and **3**, we have fit the magnetisation and susceptibility data simultaneously with PHI.^24^ In order to simplify the Hamiltonians and avoid over‐parameterisation, a three‐fold symmetric model was employed, where all three Co^II^ ions are equivalent for both **2** and **3**. Owing to the strong magnetic anisotropy, the zero‐field splitting (ZFS) of the *S=*3/2 Co^II^ ions was taken into account using a single axial *D* parameter, assuming collinear anisotropies for the three sites. In both cases we considered only isotropic exchange between all spin centres. Thus, we employed ${\hat{H}}_{\left(1\right)}$ for **2** [Eq. (1)] and ${\hat{H}}_{\left(2\right)}$ for **3** [Eq. (1)], where ${\vec{\hat{S}}}_{1}$ , ${\vec{\hat{S}}}_{2}$ and ${\vec{\hat{S}}}_{3}$ are the *S=*3/2 Co^II^ spins, ${\vec{\hat{S}}}_{4}$ is the radical *S=*1/2, $J_{1}$ is the Co‐Co exchange, $J_{2}$ is the Co‐radical exchange, D is the axial Co^II^ ZFS, $g_{Co}$ is the Co^II^ *g*‐factor, $g_{rad}$ is the radical *g*‐factor, $\mu_{B}$ is the Bohr magneton and $\vec{H}$ is the magnetic field.
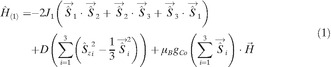

(1) $${\hat{H}}_{\left(2\right)}={\hat{H}}_{\left(1\right)}-2J_{2}\left(\sum_{i=1}^{3}{\vec{\hat{S}}}_{i}\cdot {\vec{\hat{S}}}_{4}\right)+\mu_{B\;}g_{rad}{\vec{\hat{S}}}_{4}\cdot \vec{H}$$

The magnetic data could be adequately simulated using the parameters given in Table 1, where $g_{rad}$ was fixed at 2.00. While neither simulation is perfect, the model provides good rationalisation of the magnetic data. The model can even explain the low‐temperature plateau for **2**; below 40 K the thermodynamic population is dominated by a six‐fold near‐degenerate ground state, and thus *χ*_M_ *T* becomes linear, until Zeeman depopulation occurs below 5 K. Use of rhombic ZFS with an *E* parameter did not improve the fits. The results show weak antiferromagnetic exchange between the Co^II^ sites in **2** and **3**, with coupling constants of −4.4(3) cm^−1^ and −5(1) cm^−1^ being determined using Equations (1) and (2). The Co^II^ centres and the HAN radical engage in strong antiferromagnetic interactions with *J*_2_=−290(20) cm^−1^ [Eq. (2)]. The Co^II^ *S=*3/2 ground states have significant ZFS, with the *D*‐value of −38(4) cm^−1^ for **2** being approximately double the value of −15(2) cm^−1^ for **3**. The large ZFS and *g*>2 for tetrahedral Co^II^ is consistent with significant mixing of an orbitally degenerate excited state into the ground state through spin‐orbit coupling.

**Table 1:** Parameters used for the spin Hamiltonians models of **2** and **3**.

	**2**	**3**
*g*_Co_	2.29(2)	2.15(5)
*D* [cm^−1^]	−38(4)	−15(2)
*J*_1_ [cm^−1^]	−4.4(3)	−5(1)
*J*_2_ [cm^−1^]	n/a	−290(20)

In order to verify the magnitude and the sign of *D*, we performed complete active space self‐consistent field (CASSCF) calculations on **2** and **3** using the experimental atomic coordinates. The calculations were performed with MOLCAS 8.0 (see SI for details).^25^, ^26^ To directly assess the local ZFS of the Co^II^ sites, each structurally inequivalent Co^II^ was investigated independently, where the remaining Co^II^ sites were substituted with the closed‐shell ion Zn^II^. For **3**, a singly oxidized form without the radical spin was examined using the fixed solid‐state structure to examine only the structural contribution to the ZFS. The calculations for **2** gave an average *D* value of −110(20) cm^−1^, an *E*/*D* value of approximately 0.08 and *g*_av_=2.46, while for **3** they gave an average *D* value of −57(5) cm^−1^, an *E*/*D* value of approximately 0.12 and *g*_av_=2.41. Thus, the calculations support the experimental result of both a negative *D* value with small rhombicity, as well as the trend of *D*_**2**_≈2*D*_**3**_ and *g*>2. The magnitude of *D* is overestimated in both cases by a factor of three to four, and the *g*_av_ values are higher than those found experimentally, indicating that the calculations overestimate the extent of excited state mixing into the ground state. The calculations also yield the local orientations of the magnetic anisotropy at each site. For both **2** and **3**, the easy axes of the Co^II^ ions are dictated by the local [N′′‐Co‐N′′] plane and are oriented at angles of 70–80° relative to the plane of the HAN ligands (Figure 5). Accounting for these local orientations using a non‐collinear model in PHI did not significantly enhance the quality of the fits. The different *D*‐values for **2** and **3** are likely to arise as a result of the differences in the geometric parameters associated with the individual cobalt(II) centres.

**Figure 5:**
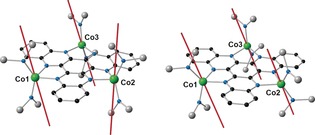
Complexes **2** (left) and **3** (right) with the orientation of the magnetic anisotropy on the individual Co^II^ sites shown as red bars. Unlabelled atoms are C (black), N (blue) and Si (grey). For clarity, hydrogen atoms and methyl groups are omitted.

Complexes of anisotropic 3d metals have stimulated huge levels of activity over the last 25 years owing to their potential single‐molecule magnet (SMM) properties.^27^, ^28^ One strategy for increasing the anisotropy barrier in an SMM is to employ strong exchange, which can lead to greater separation between the magnetic ground state and the excited states. This can be accomplished by using radical ligands.^29^, ^30^, ^31^ We were interested to see if complexes **2** and **3** displayed SMM characteristics, however measurements of the in‐phase and out‐of‐phase AC magnetic susceptibility as functions of temperature did not reveal any slow relaxation in fields of *H*_dc_=0–1500 Oe. The absence of SMM behaviour—despite the appreciable anisotropy—can be explained by the non‐zero rhombicity of the Co^II^ centres in **2** and **3**, with the irregular 4‐coordinate geometry also contributing. Similar observations on a strongly coupled, radical‐bridged dicobalt species were described recently.^32^

In conclusion, [HAN]^.−^ (**1**) was synthesized as the [K(18‐c‐6)]^+^ salt and shown to be an *S=*1/2 radical with spin density distributed across the aromatic system. Adding three equivalents of [Co(N′′)_2_] to **1** gave [K(18‐c‐6)][(HAN){Co(N′′)_2_}_3_], [K(18‐c‐6)][**3**]. The radical‐bridged complex **3** and the unreduced analogue [{(HAN){Co(N′′)_2_}_3_] (**2**) have similar molecular structures, however their magnetic properties are markedly different. In the case of **2**, weak antiferromagnetic exchange between the cobalt centres was identified, with *J*≈−4 cm^−1^ (−2 *J* formalism). In **3**, very strong coupling to the [HAN]^.−^ radical ligand was found, with *J* estimated as −290 cm^−1^. The properties of [HAN]^.−^ provide a blueprint onto which other metal ions with greater magnetic anisotropy, such as lanthanides, can be incorporated.

*In memory of Malcolm H. Chisholm FRS*

## Supporting information

As a service to our authors and readers, this journal provides supporting information supplied by the authors. Such materials are peer reviewed and may be re‐organized for online delivery, but are not copy‐edited or typeset. Technical support issues arising from supporting information (other than missing files) should be addressed to the authors.

Supplementary
