# Supplementary material for: Strong Exchange Coupling in a Trimetallic Radical‐Bridged Cobalt(II)‐Hexaazatrinaphthylene Complex
Source: Angew Chem Int Ed Engl. 2016 Mar 21;55(18):5521–5. doi: 10.1002/anie.201600694 (PMC5071680; doi:10.1002/anie.201600694)
Supplement: Supplementary file 1 — Supplementary [file ANIE-55-5521-s001.pdf]

Angewandte Chemie
Eine Zeitschrift der Gesellschaft Deutscher Chemiker

Supporting Information

Strong Exchange Coupling in a Trimetallic Radical-Bridged Cobalt(II)-Hexaazatrinaphthylene Complex

*Jani O. Moilanen, Nicholas F. Chilton, Benjamin M. Day, Thomas Pugh, and Richard A. Layfield**

anie_201600694_sm_miscellaneous_information.pdf

Contents

Synthetic details and basic characterization	1-7.
X-ray crystallography	7-8.
EPR spectroscopy and DFT calculations on [{K(18-c-6)}**1**]	8-13.
Magnetic property measurements on **2**·toluene and [K(18-c-6)][**3**]·toluene	14.
CASSCF calculations on **2** and **3**	14.
XYZ-Coordinates (B3LYP/6-311G**)	15-16.
References	17.

Synthetic details and basic characterization

General considerations. All reactions and manipulations were performed under an inert atmosphere of argon by using standard Schlenk techniques or an inert atmosphere glove box. Solvents (toluene, THF, and hexanes) were dried by refluxing either over potassium (toluene and THF) or over sodium–potassium alloy (hexanes) for at least three days before being distilled and stored over activated 4 Å molecular sieves or a potassium mirror. Solvents were degassed prior to use. THF-d_8 and benzene-d_6 were degassed and refluxed over molten potassium for three days before being degassed again and vacuum transferred into an ampoule. 18-Crown-6 was purified by sublimation. 5,6,11,12,17,18-Hexaazatrinaphthylene and $[Co(N\{SiMe_3\}_2)_2]_2$ were synthesized according to literature procedures.[1,2] Elemental analyses were carried out by Mr. Stephen Boyer at London Metropolitan University, U.K.

UV/vis/NIR spectroscopy: Electronic absorption spectra were measured on a PerkinElmer Lambda-1050 spectrometer in the range 300-2000 nm. The spectrum of [{K(18-c-6)}HAN] was measured in THF solution. The spectra of $[(HAN)\{Co(N'')_2\}_3]$ and $[K(18\text{-}c\text{-}6)][\{(HAN)\{Co(N'')_2\}_3]$ were measured as toluene solutions with approximate concentrations of 30 μM. **NMR spectroscopy**: NMR spectra were acquired on a Bruker Avance III 400 MHz spectrometer. **IR spectroscopy:** FTIR spectra were recorded on Perkin Elmer Spectrum RX1 Spectrometer as Nujol mulls in KBr discs.

Synthesis of [{K(18-c-6)}HAN] ([{K(18-c-6)}1]). A mixture of HAN (562 mg, 1.46 mmol), 18-crown-6 (389 mg, 1.47 mmol) and potassium (57 mg, 1.46 mmol) was vigorously stirred for five hours in toluene (50 ml) at 40°C, during which time the mixture changed color from yellow to dark red. The red mixture was allowed cool to ambient temperature and stirred overnight. The solvent was removed under vacuum and the remaining red solid was washed with hexanes (2 × 20 ml), giving [{K(18-c-6)}**1**] as a red powder (902 mg, 90%). Elemental analysis (%) calcd. for $C_{36}H_{36}KN_6O_6$: C 62.86, H 5.28, N 12.22; found: C 62.79, H 5.32, N 12.15. ^{1}H NMR (400.07 MHz, THF-d_8, 296 K, δ/ppm) = 3.57 (s, 18-c-6). IR ($\tilde{\nu}$/cm^{-1}): 1549 (w), 1330 (m), 1284 (m), 1192 (w), 1118 (m), 1076 (w), 952 (w), 895 (w), 805 (w), 767 (m), 756 (m), 720 (m), 601 (m).

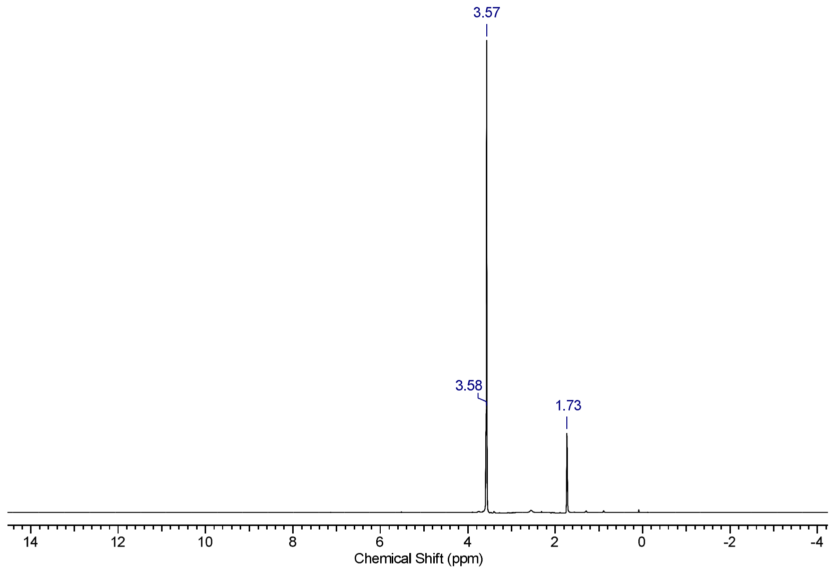

Figure S1. ^{1}H NMR spectrum of [{K(18-c-6)}**1**] in THF-d_8.

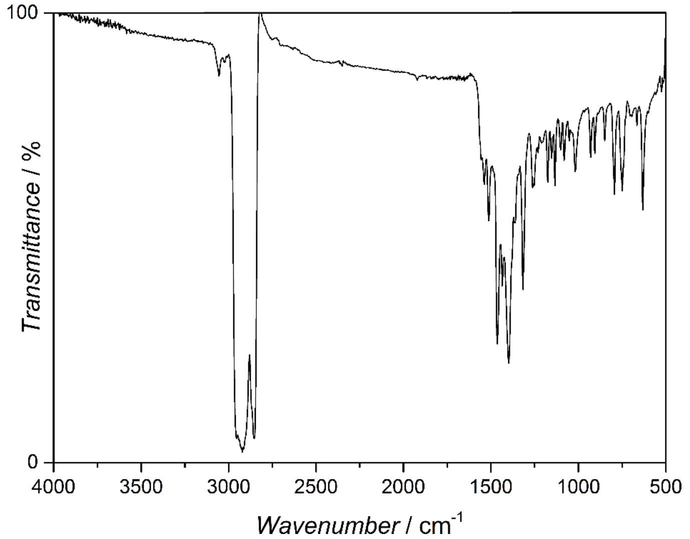

Figure S2. IR spectrum of [{K(18-c-6)}**1**] as a Nujol mull in KBr discs.

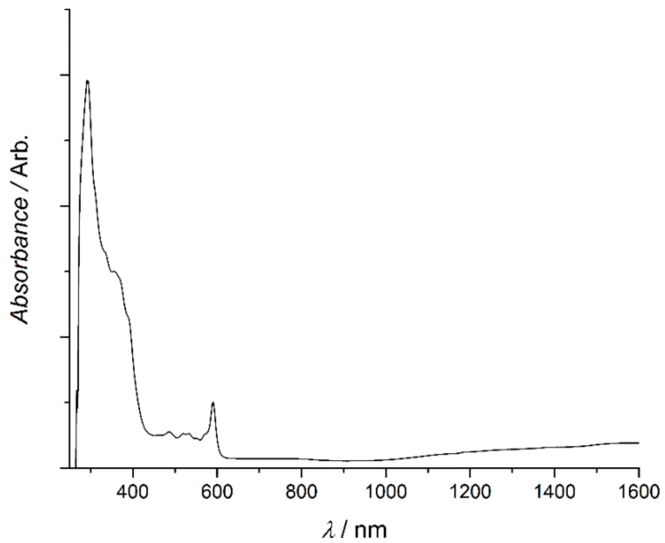

Figure S3. Electronic absorption spectrum of [{K(18-c-6)}**1**] in THF.

Synthesis of [{(HAN)Co(N″)$_2$}$_3$]·toluene (2·toluene). [Co(N{SiMe$_3$}$_2$)$_2$]$_2$ (357 mg, 0.470 mmol) and HAN (120 mg, 0.312 mmol) were stirred in toluene (17 ml) at room temperature for three hours, producing a green-black solution. The solution was filtered, concentrated and stored at +4°C overnight, which resulted in the formation of **2**·toluene as black needle-like crystals (158 mg, 33% isolated yield). ^{1}H NMR (400.07 MHz, toluene-d$_8$, 296 K, δ/ppm): 11.71 (6H, HAN-H), 9.90 (6H, HAN-H), 8.70 (108H, 12 × SiMe$_3$). Elemental analysis (%) calcd. for $C_{60}H_{120}Co_3N_{12}Si_{12}$: C 47.30, H 7.94, N 11.03; found: C 47.25, H 7.86, N 10.95. IR ($\tilde{\nu}$/cm^{-1}): 1608 (w), 1558 (w), 1519 (w), 1243 (m/s), 1180 (w), 1138 (w), 1087 (w), 932 (m), 743 (s), 779 (m), 755 (m), 663 (m), 623 (w), 605 (w).

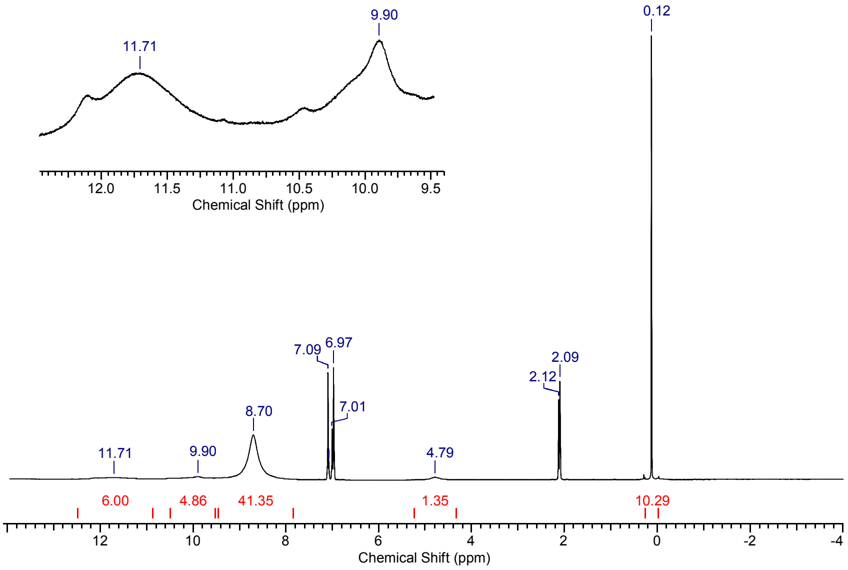

Figure S4. ^{1}H NMR spectrum of **2**·toluene in toluene-d_8.

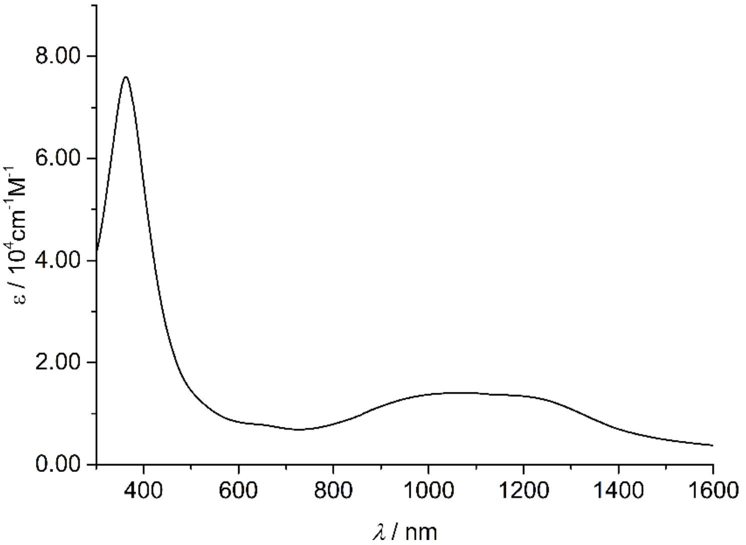

Figure S5. Electronic absorption spectrum of **2**·toluene in toluene (λ = 362 nm and 1067 nm).

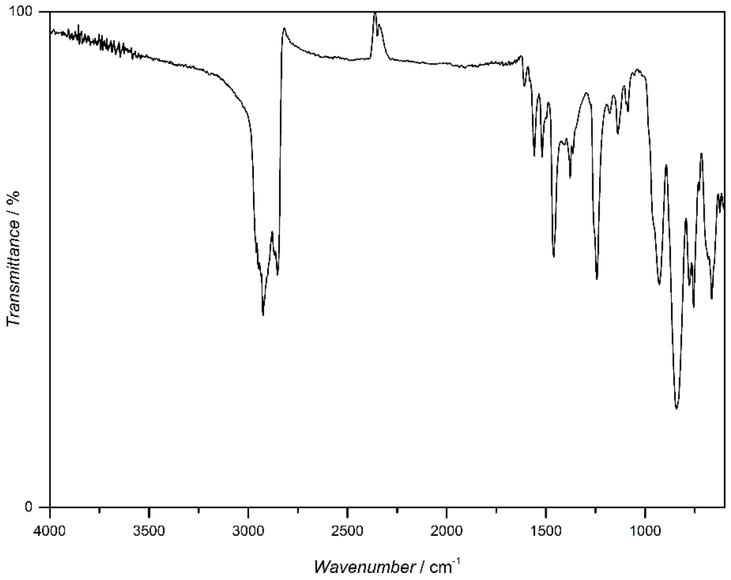

Figure S6. IR spectrum of **2**·toluene as a Nujol mull in KBr discs.

Synthesis of [{K(18-c-6)}{(HAN)Co(N″)$_2$}$_3$]·toluene ([K(18-c-6)][3]·toluene). A solution of [Co(N{SiMe$_3$}$_2$)$_2$]$_2$ (168 mg, 0.22 mmol) in toluene (10 ml) was added dropwise to a dark red suspension of [HAN][K(18-crown-6)] (100 mg, 0.145 mmol) in toluene (10 ml) at 0°C. During the addition the solution turned black and a small amount of black solid precipitated from the solution. The reaction mixture was warmed to room temperature and stirred for two days. The black mixture was filtered, concentrated and the filtrate was stored at +4°C for two weeks, which produced [K(18-c-6)][**3**]·toluene as black crystals (118 mg, 45 %). ^{1}H NMR (400.07 MHz, toluene-d$_8$, 296 K, δ/ppm) 8.24 (12H, HAN-H), 6.20 (108H, 12 × SiMe$_3$), 4.84 (24H, 18-c-6). Elemental analysis (%) calcd. for $C_{72}H_{144}Co_3KN_{12}O_6Si_{12}$: C 47.34, H 7.94, N 9.20; found: C: 47.19, H 7.81, N 9.18. IR ($\tilde{\nu}$/cm^{-1}): 1560 (w), 1509 (w), 1495 (w), 1419 (w), 1352 (w), 1237 (m), 1133 (m), 1109 (m), 1094 (m), 962 (s), 864 (s), 843 (s), 826 (s), 779 (m), 758 (m), 704 (w), 657 (w), 607 (w).

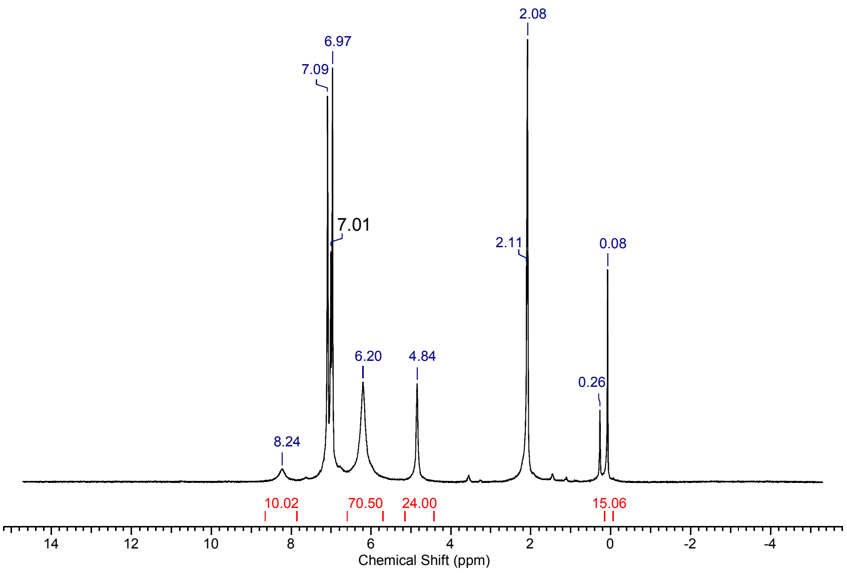

Figure S7. ^{1}H NMR spectrum of [K(18-c-6)][**3**]·toluene in toluene-d_8.

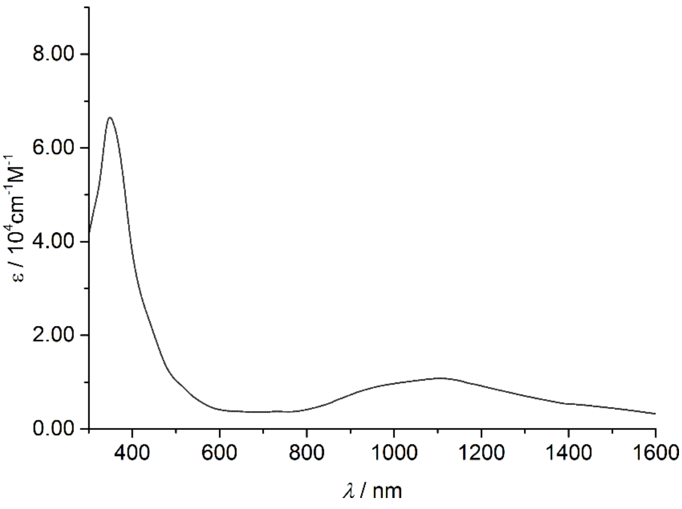

Figure S8. Electronic absorption spectrum of [K(18-c-6)][**3**]·toluene (λ = 349 nm and 1108 nm).

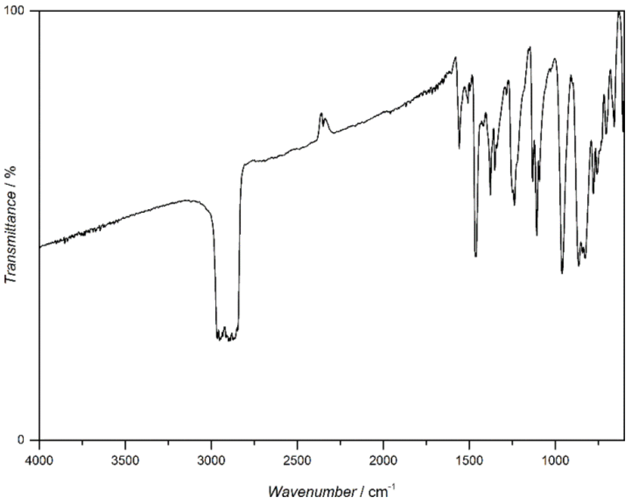

Figure S9. IR spectrum of [K(18-c-6)][**3**]·toluene as a Nujol mull in KBr discs.

X-ray crystallography

X-ray diffraction data for **2**·toluene were collected using a Bruker APEX-II diffractometer with CuKα radiation. X-ray diffraction data for [K(18-c-6)][**3**]·toluene were collected on an Agilent Technologies SuperNova diffractometer using MoKα radiation. Structures were solved with SHELXS using direct methods and refined with SHELXL using least-squares minimization.[3] For [K(18-c-6)][**3**]·toluene four *bis*(trimethylsilyl)amide groups, three methyl groups, and 18-crown-6 coordinated were refined over two positions with occupancy ratios of 0.74:0.26, 0.74:0.26, 0.61:0.39, 0.61:0.39, 0.60:0.40, 0.80:0.20, 0:61:0.39 and 0.55:0.45, respectively. In addition to this, the toluene solvent molecules were refined over two positions with the occupancy ratios 0.50:0.50 and 0:68:0.32.

Table S1. Crystal data and structural refinement for **2**·toluene and [K(18-c-6)][**3**]·toluene.

	**2·toluene**	**[K(18-c-6)][3]·2toluene**
CCDC ref. code	1448294	1448295
Formula	$C_{67}H_{128}Co_3N_{12}Si_{12}$	$C_{86}H_{160}Co_3KN_{12}O_6Si_{12}$
FW	1615.68	2011.22
Crystal system	monolinic	Triclinic
Space group	$P2_1/c$	$P\bar{1}$
a/Å	11.9754(2)	16.9481(6)
b/Å	22.9253(4)	18.5033(6)
c/Å	32.2034(5)	20.6309(7)
α/°	90.0	90.998(3)
β/°	97.001(2)	105.055(3)
γ/°	90	112.378(3)
V/Å^3	8775.2(3)	5728.0(4)
Z	4	2
Crystal size/mm^3	0.2 × 0.1 × 0.1	0.7 × 0.25 × 0.09
2θ range/°	4.744 to 133.19	6.494 to 50.0000
Reflections collected	32032	56116
Independent reflections, R_{int}	14799, 0.0470	20117, 0.0445
Completeness/%	95.4	99.9
Data/restraints/parameters	14799/2/884	20117/544/1459
Goodness-of-fit on F^2	1.027	1.019
Final R indices [I>2σ(I)]	R_1 = 0.0572	R_1 = 0.0514
	wR_2 = 0.1455	wR_2 = 0.1179
R indices (all data)	R_1 = 0.0785	R_1 = 0.0913
	wR_2 = 0.1557	wR_2 = 0.1395

EPR spectroscopy and DFT calculations on [{K(18-c-6)}1]

The CW X-band spectrum of [{K(18-c-6)}**1**] was recorded as a solid (powder) and a solution (< 1 × 10^{-4} M in THF) on a Bruker EMX Micro X-Band spectrometer at 293 K. The exact concentration of THF solution of [HAN][K(18-crown-6)] could not be determined due its poor solubility in THF or other organic solvents. The experimental g_{iso} values 2.0072 G (powder) and 2.0070 G (THF) were corrected with respect to the Bruker strong pitch (2.0028 G) to give g_{iso} values of 2.0033 G and 2.0033 G for the powder and solution samples, respectively.

A simulation for the solution-phase EPR spectrum of [{K(18-c-6)}**1**] was performed with the EasySpin toolbox for Matlab.[4] The microwave frequency (9.464 MHz) and modulation amplitude (0.0025 mT) employed on the simulation were obtained from experimental settings. A pure Lorentzian peak-to-peak linewidth (0.01 mT), g_{iso} = 2.0072 G and the following set of isotropic hyperfine coupling constants (IHCCs) were used in simulation (in MHz): $^{14}N_{1\text{-}6}$ = 3.65, $^{1}H_{1,4,5,8,9,12}$ = –1.84, $^{1}H_{2,3,6,7,10,11}$ = –1.83, K = 3.67, $C_{1\text{-}6}$ = –5.47, $C_{7,12,13,18,19,24}$ = –5.15, $C_{8,11,14,17,20,23}$ = 0.75 (atom numbers shown in Figure S10e). Although a reasonable simulation was obtained only by using the IHCCs of ^{14}N and ^{1}H nucleus, the inclusion of IHCCs of C nucleus into the simulation was necessary in order to produce the experimentally detected satellite lines. A small improvement in the simulation was obtained by including K in the simulation, although the differences in the simulation with and without potassium are too small to be regarded as diagnostic, although an interaction between the HAN radical and the $[K(18\text{-crown-}6)]^+$ cation is possible. Similar interactions have been observed for analogous ion pairs consisting of organic monoanion radical and alkali metal counter cation both in a solid state and solution phase.[5] Additional support for the cation-anion interaction in [{K(18-c-6)}**1**] was obtained from the Mulliken population analysis which shows that a small amount of spin density is found on potassium (see Table SX).

DFT calculation details: all calculations were done using Gaussian 09[6] and Turbomole 6.3[7] program packages, and visualization of molecular orbitals and spin densities employed Gview.[8] The geometries of $[HAN]^{\bullet-}$ and of [{K(18-c-6)}**1**] were fully optimized in D_{3h} and C_3, point groups, respectively, using the B3LYP[9] exchange-correlation functional combined with either 6-311G**[10] or def2-TZVP[11] triple-ζ valence quality basis set. For [{K(18-c-6)}**1**] different geometries were used as a starting guess in optimizations to ensure that the global minimum structure was found (Figure S12). Frequency analyses were performed for both optimized structures to ensure that they correspond to true minima (no imaginary frequencies) on the potential energy hypersurface.

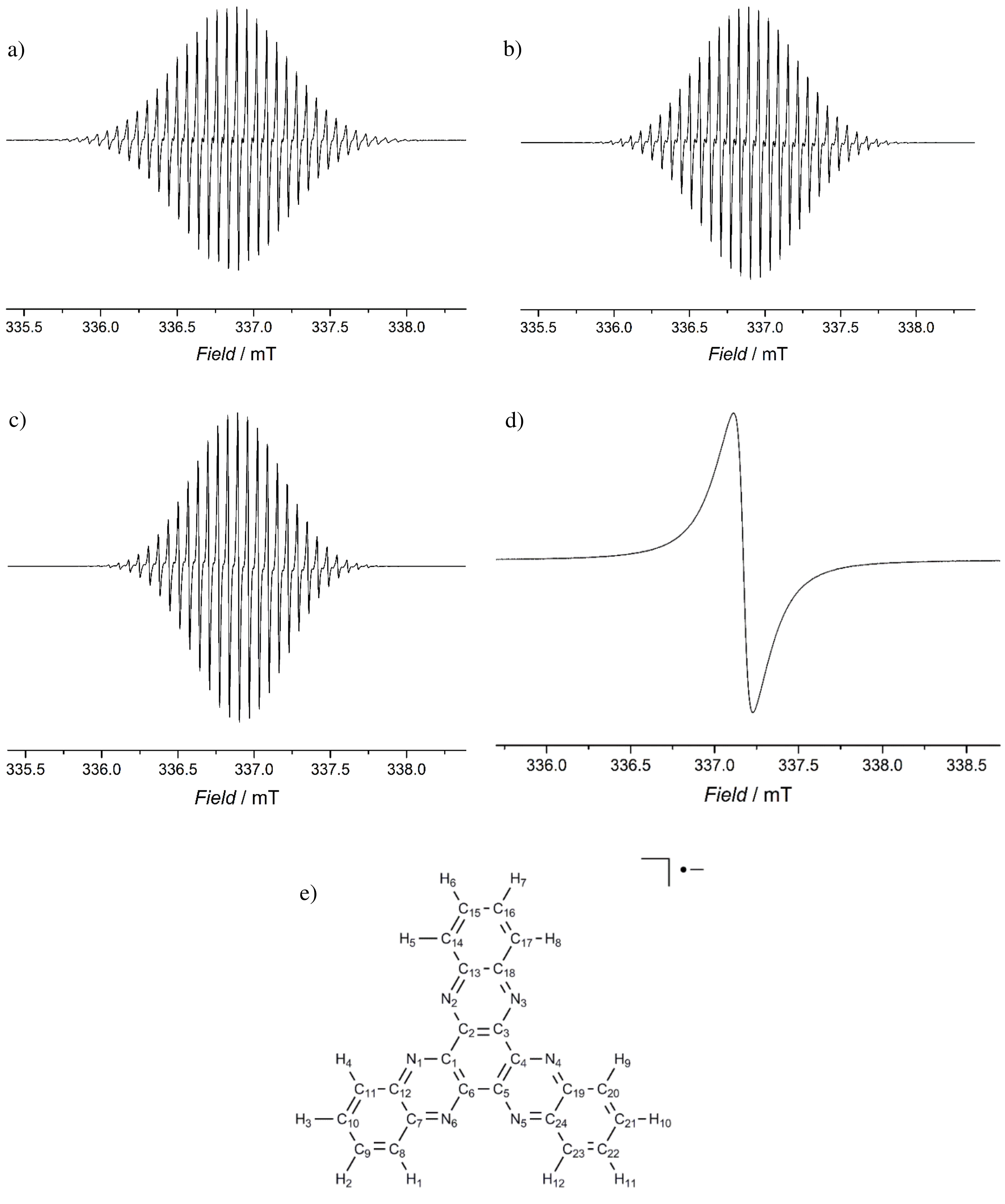

Figure S10. a) Solution-state X-band EPR spectrum of [{K(18-c-6)}**1**] at 293 K in THF (mod. amp.= 0.025 G, g_{iso} = 2.0072 G, corrected g_{iso} = 2.0033) G. **b)** Simulated EPR spectrum of [{K(18-c-6)}**1**] (mod. amp.= 0.025 G, lw = 0.01 mT, g_{iso} = 2.0072). IHCC/MHz: $^{14}N_{1\text{-}6}$ = 3.65, $^{1}H_{1,4,5,8,9,12}$ = –1.84, $^{1}H_{2,3,6,7,10,11}$ = –1.83, K = 3.67, $C_{1\text{-}6}$ = –5.47, $C_{7,12,13,18,19,24}$ = –5.15, $C_{8,11,14,17,20,23}$ = 0.75. **c)** Simulated EPR spectrum of [{K(18-c-6)}**1**] without K (mod. amp.= 0.025 G, lw = 0.01 mT, g_{iso} = 2.0072) IHCC/MHz: $^{14}N_{1\text{-}6}$ = 3.65, $^{1}H_{1,4,5,8,9,12}$ = –1.85, $^{1}H_{2,3,6,7,10,11}$ = –1.84, $C_{1\text{-}6}$ = –5.42, $C_{7,12,13,18,19,24}$ = –5.06, $C_{8,11,14,17,20,23}$ = 0.76. **d)** Powder X-band EPR spectrum of [{K(18-c-6)}**1**] 293 K (mod. amp. = 0.01 G, lw = 0.14 mT, g_{iso} = 2.0072 G, corrected g_{iso} = 2.0033 G). **e)** Atom numbering scheme in [{K(18-c-6)}**1**].

Table S2. Simulated and calculated (B3LYP) isotropic hyperfine coupling constants (in MHz) for $[HAN]^{\bullet-}$ and [{K(18-c-6)}**1**].

			$[HAN]^{\bullet-}$		[{K(18-c-6)}1]	
**Nucleus**[a]	**Simulation 1**[b]	**Simulation 2**[c]	**6-311G****	**def2-TZVP**	**6-311G****	**def2-TZVP**
$^{14}N_{1-6}$	3.65	3.65	3.76	4.06	3.81	4.08
$^{1}H_{1,4,5,8,9,12}$	-1.84	-1.85	-2.61	-2.41	-2.10	-1.96
$^{1}H_{2,3,6,7,10,11}$	-1.83	-1.84	-1.66	-1.70	-1.73	-1.77
K	3.67	-	-	-	1.50	1.83
C_{1-6}	-5.47	-5.42	-5.16	-4.90	-4.94	-4.91
$C_{7,12,13,18,19,24}$	-5.15	-5.07	-4.98	-4.89	-4.70	-4.48
$C_{8,11,14,17,20,23}$	0.75	0.76	1.19	1.31	0.53	0.64
$C_{9,10,15,16,21,22}$	-[d]	-[d]	-0.48	-0.29	-0.20	-0.05

[a] Atom numbers in Figure S10e.

[b] Simulated with K.

[c] Simulated without K.

[d] Not included in simulations.

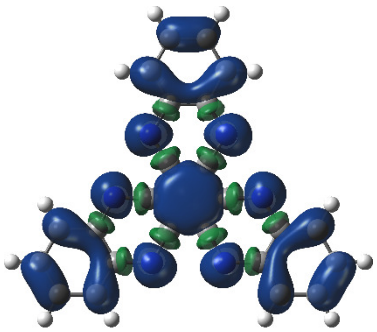

Figure S11. Spin density for $[HAN]^{\bullet-}$ calculated at the B3LYP/6-311G** level. Blue = α spin density; green = β spin density. The distribution of spin density in [{K(18-c-6)}**1**] is essentially the same.

Table S3. B3LYP Mulliken spin densities for $[HAN]^{\bullet-}$ and [{K(18-c-6)}**1**].

	$[HAN]^{\bullet-}$		[{K(18-c-6)}1]	
**Atom**	**6-311****	**def-TZVP2**	**6-311****	**def-TZVP2**
K	-	-	0.003	0.004
N_1	0.120	0.118	0.121	0.117
N_2	0.120	0.118	0.121	0.118
N_3	0.120	0.118	0.121	0.117
N_4	0.120	0.118	0.121	0.118
N_5	0.120	0.118	0.121	0.117
N_6	0.120	0.118	0.121	0.118
C_1	-0.003	-0.001	-0.005	-0.002
C_2	-0.003	-0.001	-0.005	-0.002
C_3	-0.003	-0.001	-0.005	-0.002
C_4	-0.003	-0.001	-0.005	-0.002
C_5	-0.003	-0.001	-0.005	-0.002
C_6	-0.003	-0.001	-0.005	-0.002
C_7	-0.004	-0.001	0.002	0.005
C_8	0.038	0.032	0.029	0.024
C_9	0.020	0.022	0.022	0.023
C_{10}	0.020	0.022	0.022	0.023
C_{11}	0.038	0.032	0.029	0.024
C_{12}	-0.004	-0.001	0.002	0.005
C_{13}	-0.004	-0.001	0.002	0.005
C_{14}	0.038	0.032	0.029	0.024
C_{15}	0.020	0.022	0.022	0.023
C_{16}	0.020	0.022	0.022	0.023
C_{17}	0.038	0.032	0.029	0.024
C_{18}	-0.004	-0.001	0.002	0.005
C_{19}	-0.004	-0.001	0.002	0.005
C_{20}	0.038	0.032	0.029	0.024
C_{21}	0.020	0.022	0.022	0.023
C_{22}	0.020	0.022	0.022	0.023
C_{23}	0.038	0.032	0.029	0.024
C_{24}	-0.004	-0.001	0.002	0.005
H_1	-0.003	-0.002	-0.002	-0.002
H_2	-0.002	-0.001	-0.002	-0.001
H_3	-0.002	-0.001	-0.002	-0.001
H_4	-0.003	-0.002	-0.002	-0.002
H_5	-0.003	-0.002	-0.002	-0.002
H_6	-0.002	-0.001	-0.002	-0.001
H_7	-0.002	-0.001	-0.002	-0.001
H_8	-0.003	-0.002	-0.002	-0.002
H_9	-0.003	-0.002	-0.002	-0.002
H_{10}	-0.002	-0.001	-0.002	-0.001
H_{11}	-0.002	-0.001	-0.002	-0.001
H_{12}	-0.003	-0.002	-0.002	-0.002

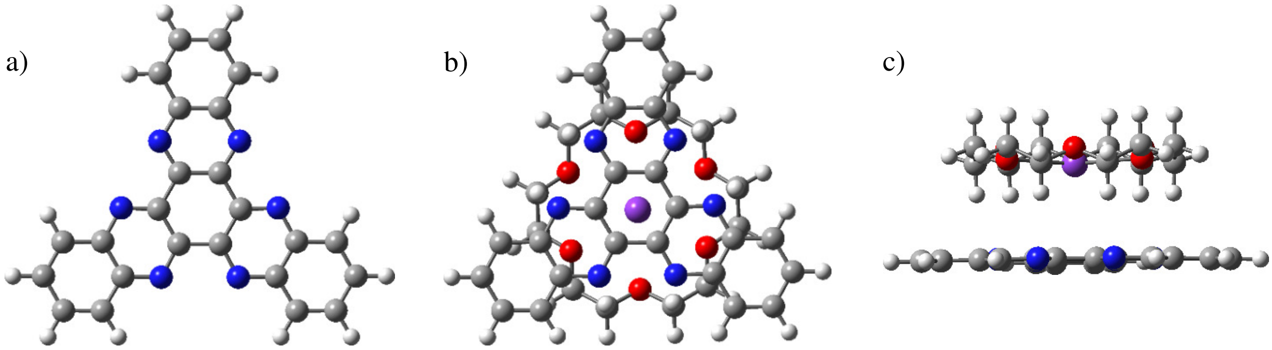

Figure S12. B3LYP/6-311G** optimized geometries of: (a) $[HAN]^{\bullet -}$ and; (b), (c) [{K(18-c-6)}**1**]. Grey = C; blue = N; red = O; white = H; purple = K.

Table S4. Selected bond lengths (Å) for $[HAN]^{\bullet -}$ and [{K(18-c-6)}**1**] calculated at the B3LYP/6-311G** and B3LYP/def2-TZVP levels of theory.

	$[HAN]^{\bullet -}$		[{K(18-c-6)}1]	
**Bond**	**6-311G****	**def2-TZVP**	**6-311G****	**def2-TZVP**
N_1-C_1	1.328	1.324	1.329	1.325
N_2-C_2	1.328	1.324	1.329	1.325
N_3-C_3	1.328	1.324	1.329	1.325
N_4-C_4	1.328	1.324	1.329	1.325
N_5-C_5	1.328	1.324	1.329	1.325
N_6-C_6	1.328	1.324	1.329	1.325
N_1-C_{12}	1.359	1.356	1.360	1.357
N_2-C_{13}	1.359	1.356	1.360	1.357
N_3-C_{18}	1.359	1.356	1.360	1.357
N_4-C_{19}	1.359	1.356	1.360	1.357
N_5-C_{24}	1.359	1.356	1.360	1.357
N_6-C_7	1.359	1.356	1.360	1.357
C_1-C_2	1.473	1.470	1.473	1.470
C_2-C_3	1.434	1.431	1.433	1.430
C_3-C_4	1.473	1.470	1.473	1.470
C_4-C_5	1.434	1.431	1.433	1.430
C_5-C_6	1.473	1.470	1.473	1.470
C_6-C_1	1.434	1.431	1.433	1.430
C_7-C_{12}	1.430	1.427	1.429	1.426
C_{13}-C_{18}	1.430	1.427	1.429	1.426
C_{19}-C_{24}	1.430	1.427	1.429	1.426

Magnetic property measurements

The magnetic properties of polycrystalline samples of **2**·toluene and [K(18-c-6)][**3**]·toluene were measured using a Quantum Design MPMS-7 SQUID magnetometer. The temperature dependence of magnetic susceptibility (χ) was measured at various temperatures in the range 2-300 K, and the field (H) dependence of the magnetization (M) was measured at T = 1.8, 3.0 and 5.0 K at various fields in the range 0-7 T. Samples of **2**·toluene and [K(18-c-6)][**3**]·toluene were prepared in a glove-box by adding crushed crystalline material to borosilicate NMR tubes. Eicosane (ca. 50% of the sample weight) was added and the NMR tubes were flame sealed *in vacuo*: the eicosane was melted at 50°C until the sample was covered to prevent torqueing of the crystallites and to provide good thermal contact between the sample and the bath. The plots of $\chi_M T(T)$ in a field of 1 T, and plots of $M(H)$ for both compounds are shown in Figure 2 of the main manuscript.

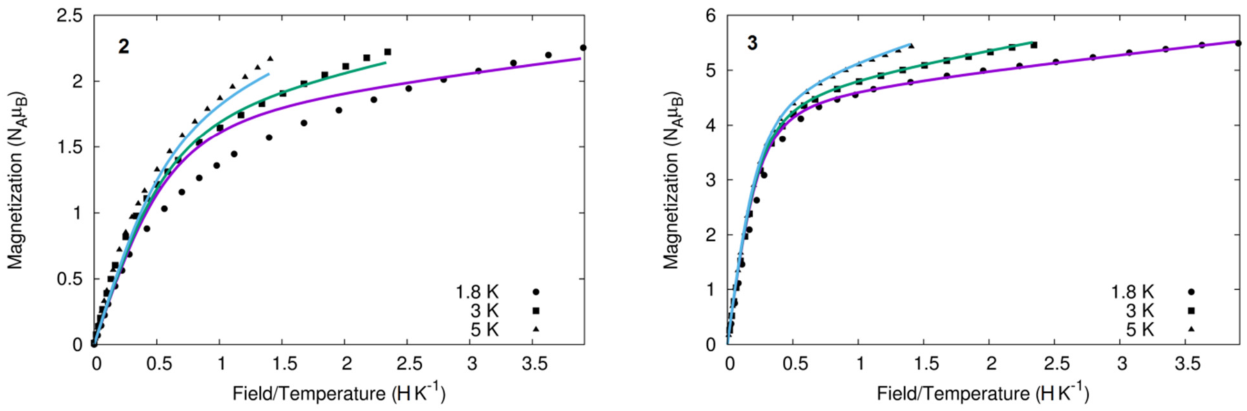

Figure S13. Reduced magnetization data for **2**·toluene and [K(18-c-6)][**3**]·toluene.

CASSCF calculations on 2 and 3

In order to verify the magnitude and the sign of D, we performed Complete Active Space Self-Consistent Field (CASSCF) calculations on **2** and **3** using the experimental atomic coordinates. An active space of seven electrons in the five 3d orbitals was used. The 10 S = 3/2 and 40 S = 1/2 configurations were considered both in the orbital optimisation (RASSCF) and the spin-orbit mixing (RASSI) procedures, and the ZFS of the S = 3/2 ground state was extracted from the spin-orbit mixed states (SINGLE_ANISO).

XYZ-Coordinates (B3LYP/6-311G)**

HAN$^{\bullet-}$

E = -1250.66485191

Number of imaginary frequencies (NIMAG) = 0

C	0.716797	1.263992	0.000000
C	-0.716797	1.263992	0.000000
C	1.453047	-0.011231	0.000000
C	0.736250	-1.252760	0.000000
C	-0.736250	-1.252760	0.000000
C	-1.453047	-0.011231	0.000000
N	1.362509	-2.423574	0.000000
C	2.721283	-2.396808	0.000000
C	3.436338	-1.158296	0.000000
N	2.780131	0.031819	0.000000
N	-1.362509	-2.423574	0.000000
C	-2.721283	-2.396808	0.000000
C	-3.436338	-1.158296	0.000000
N	-2.780131	0.031819	0.000000
N	-1.417622	2.391754	0.000000
N	1.417622	2.391754	0.000000
C	0.715055	3.555104	0.000000
C	-0.715055	3.555104	0.000000
C	-3.447624	-3.610823	0.000000
C	-4.850877	-1.180319	0.000000
C	-1.403252	4.791142	0.000000
C	1.403252	4.791142	0.000000
C	4.850877	-1.180319	0.000000
C	3.447624	-3.610823	0.000000
C	4.829075	-3.602311	0.000000
C	5.534230	-2.380946	0.000000
C	-0.705155	5.983257	0.000000
C	0.705155	5.983257	0.000000
C	-4.829075	-3.602311	0.000000
C	-5.534230	-2.380946	0.000000
H	-2.877943	-4.533298	0.000000
H	-5.364923	-0.225723	0.000000
H	-2.486980	4.759021	0.000000
H	2.486980	4.759021	0.000000
H	5.364923	-0.225723	0.000000
H	2.877943	-4.533298	0.000000
H	-5.376828	-4.539283	0.000000
H	-6.619548	-2.386828	0.000000
H	5.376828	-4.539283	0.000000
H	6.619548	-2.386828	0.000000
H	1.242720	6.926111	0.000000
H	-1.242720	6.926111	0.000000

[HAN][K(18-crown-6)]

E = -2773.88905943

NIMAG = 0

K	0.000000	0.000000	1.730273
O	-1.381632	2.428655	1.915951
O	1.429645	2.440205	2.280568
O	2.794093	-0.017799	1.915951
O	1.398457	-2.458211	2.280568
O	-1.412460	-2.410856	1.915951
O	-2.828102	0.018007	2.280568
C	-0.672344	3.582503	2.352682
H	-0.623810	3.598924	3.451138
H	-1.186975	4.491617	2.013097
C	0.720211	3.568597	1.769470
H	0.680163	3.524339	0.675060
H	1.231153	4.497241	2.063655
C	2.762614	2.372547	1.774524
H	3.322295	3.271944	2.070720
H	2.747174	2.315571	0.680232
C	3.452601	1.163397	2.358781
H	4.498870	1.159387	2.024208
H	3.438093	1.211294	3.457303
C	3.438711	-1.208984	2.352682
H	3.428665	-1.259227	3.451138
H	4.483342	-1.217858	2.013097

C	2.730390	-2.408020	1.769470
H	2.712085	-2.351208	0.675060
H	3.279148	-3.314830	2.063655
C	0.673379	-3.578768	1.774524
H	1.172439	-4.513164	2.070720
H	0.631757	-3.536908	0.680232
C	-0.718769	-3.571739	2.358781
H	-1.245376	-4.475829	2.024208
H	-0.670035	-3.583123	3.457303
C	-2.766366	-2.373519	2.352682
H	-2.804855	-2.339697	3.451138
H	-3.296367	-3.273759	2.013097
C	-3.450601	-1.160577	1.769470
H	-3.392249	-1.173131	0.675060
H	-4.510301	-1.182410	2.063655
C	-3.435993	1.206220	1.774524
H	-4.494734	1.241220	2.070720
H	-3.378931	1.221337	0.680232
C	-2.733832	2.408342	2.358781
H	-3.253493	3.316442	2.024208
H	-2.768058	2.371829	3.457303
C	-1.252471	-0.736461	-1.653701
C	-0.011556	-1.452901	-1.654969
C	-1.252471	0.736458	-1.654969
C	-0.011558	1.452902	-1.653701
C	1.264027	0.716443	-1.654969
C	1.264030	-0.716441	-1.653701
N	0.029017	2.780957	-1.642399
C	-1.160828	3.439711	-1.669037
C	-2.398291	2.725215	-1.672244
N	-2.422873	1.365319	-1.646908
N	2.393837	1.415610	-1.646908
C	3.559251	0.714373	-1.672244
C	3.559291	-0.714549	-1.669037
N	2.393871	-1.415608	-1.642399
N	0.029036	-2.780929	-1.646908
N	-2.422888	-1.365349	-1.642399
C	-2.398463	-2.725162	-1.669037
C	-1.160960	-3.439589	-1.672244
C	4.793937	1.404145	-1.691198
C	4.794107	-1.404065	-1.684419
C	-1.180943	-4.853743	-1.691198
C	-3.613010	-3.449786	-1.684419
C	-3.612993	3.449599	-1.691198
C	-1.181097	4.853851	-1.684419
C	-2.381629	5.535668	-1.702941
C	-3.603214	4.830278	-1.706501
C	-2.381536	-5.535614	-1.706501
C	-3.603214	-4.830385	-1.702941
C	5.984750	0.705336	-1.706501
C	5.984843	-0.705283	-1.702941
H	4.767332	2.487722	-1.709687
H	4.767745	-2.487725	-1.698050
H	-0.229235	-5.372491	-1.709687
H	-4.538305	-2.885126	-1.698050
H	-4.538097	2.884769	-1.709687
H	-0.229439	5.372851	-1.698050
H	6.927147	1.241372	-1.730976
H	6.927281	-1.241221	-1.724774
H	-2.388712	6.619812	-1.724774
H	-4.538633	5.378400	-1.730976
H	-4.538569	-5.378591	-1.724774
H	-2.388514	-6.619771	-1.730976

References

1. S. Barlow, Q. Zhang, B. R. Kaafarani, C. Risko, F. Amy, C. K. Chan, B. Domercq, Z. A. Starikova, M. Y. Antipin, T. V. Timofeeva, B. Kippelen, J.-L. Brédas, A. Kahn, S. R. Marder, *Chem. Eur. J.* **2007**, *13*, 3537.
2. A. M. Bryan, G. J. Long, F. Grandjean, P. P. Power, *Inorg. Chem.* **2013**, *52*, 12152.
3. G. M. Sheldrick, *Acta Crystallogr., Sect. A* **2008**, *A64*, 112.
4. S. Stoll, A. Schweiger, *J. Magn. Reson.* **2006**, *178*, 42. (b) MATLAB version R2014a. Natick, Massachusetts: The MathWorks Inc., 2014.
5. (a) A. V. Zabula, S. N. Spisak, A. S. Filatov, V. M. Grigoryants, M. A. Petrukhina, *Chem. Eur. J.* **2012**, *18*, 6476. (b) J.-M. Lü, S. V. Rosokha, S. V. Lindeman, I. S. Neretin, J. K. Kochi, *J. Am. Chem. Soc.* **2005**, *127*, 1797. (c) C. Dutan, S. Choua, T. Berclaz, M. Geoffroy, N. Mézailles, A. Moores, L. Ricard, P. Le Floch, *J. Am. Chem. Soc.* **2003**, *125*, 4487. (d) P. B. Hitchcock, M. F. Lappert, A. V. Protchenko, *J. Am. Chem. Soc.* **2001**, *123,* 189. (e) K. S. Chen, J. K. S. Wan, *J. Am. Chem. Soc.* **1978**, *100*, 6051. (f) G. V. Nelson, A. Von Zelewsky, *J. Am. Chem. Soc.* **1975**, *97*, 6279.
6. *Gaussian 09*, Revision D.01, M. J. Frisch, G. W. Trucks, H. B. Schlegel, G. E. Scuseria, M. A. Robb, J. R. Cheeseman, G. Scalmani, V. Barone, B. Mennucci, G. A. Petersson, H. Nakatsuji, M. Caricato, X. Li, H. P. Hratchian, A. F. Izmaylov, J. Bloino, G. Zheng, J. L. Sonnenberg, M. Hada, M. Ehara, K. Toyota, R. Fukuda, J. Hasegawa, M. Ishida, T. Nakajima, Y. Honda, O. Kitao, H. Nakai, T. Vreven, J. A. Montgomery, Jr., J. E. Peralta, F. Ogliaro, M. Bearpark, J. J. Heyd, E. Brothers, K. N. Kudin, V. N. Staroverov, R. Kobayashi, J. Normand, K. Raghavachari, A. Rendell, J. C. Burant, S. S. Iyengar, J. Tomasi, M. Cossi, N. Rega, J. M. Millam, M. Klene, J. E. Knox, J. B. Cross, V. Bakken, C. Adamo, J. Jaramillo, R. Gomperts, R. E. Stratmann, O. Yazyev, A. J. Austin, R. Cammi, C. Pomelli, J. W. Ochterski, R. L. Martin, K. Morokuma, V. G. Zakrzewski, G. A. Voth, P. Salvador, J. J. Dannenberg, S. Dapprich, A. D. Daniels, Ö. Farkas, J. B. Foresman, J. V. Ortiz, J. Cioslowski, D. J. Fox, Gaussian, Inc., Wallingford CT, 2009.
7. *TURBOMOLE V6.6 2014*; University of Karlsruhe and Forschungszentrum Karlsruhe GmbH: Karlsruhe, Germany, 1989−2007; TURBOMOLE GmbH: Karlsruhe, Germany, 2007. Available from http://www.turbomole.com.
8. GaussView, Version 5.0.8, R. Dennington, T. Keith, J. Millam, *Semichem Inc.*, Shawnee Mission, KS, 2009.
9. (a) A. D. Becke, *Phys. Rev. A: At., Mol., Opt. Phys.* **1988**, *38*, 3098. (b) C. Lee, W. Yang, R. G. Parr, *Phys. Rev. B: Condens. Matter Mater. Phys.* **1988**, *37*, 785. (c) P. J. Stephens, F. J. Devlin, C. F. Chabalowski, M. J. J. Frisch, *Phys. Chem.* **1994**, *98*, 11623.
10. (a) R. Krishnan, J.S. Binkley, R. Seeger, J.A. Pople, *J. Chem. Phys.* **1980**, *72*, 650. (b) A.D. McLean, G.S. Chandler *J. Chem. Phys.* **1980**, *72*, 5639. (c) J-P. Blaudeau, M. P. McGrath, L.A. Curtiss, L. Radom, *J. Chem. Phys.* **1997**, *107*, 5016.
11. F. Weigend, R. Ahlrichs, *Phys. Chem. Chem. Phys.* **2005**, *7*, 3297.
